# Thermal reaction characteristics of dioxins on cement kiln dust

**DOI:** 10.1039/c7ra09220b

**Published:** 2018-01-17

**Authors:** Ming-Xiu Zhan, Shuping Pan, Ivan Deviatkin, Tong Chen, Xiao-Dong Li

**Affiliations:** College of Metrology and Measurement Engineering, China Jiliang University Hangzhou 310018 China; Zhejiang Province Environmental Monitoring Centre Hangzhou 310012 China; Lappeenranta University of Technology, Sustainability Science P.O. Box 20 FI-53851 Lappeenranta Finland; State Key Laboratory of Clean Energy Utilization, Institute for Thermal Power Engineering, Zhejiang University Hangzhou Zhejiang 310027 China chentong@zju.edu.cn

## Abstract

Cement kiln dust is commonly recycled back into the production process. This results in elevated concentrations of polychlorinated dibenzo-*p*-dioxins and dibenzofurans (PCDD/Fs) in the flue gases of cement plants. The present study investigated the effects the reaction temperature, oxygen content, and origin of kiln dust had on the thermal reaction characteristics of PCDD/Fs. The concentration of 2,3,7,8-PCDD/Fs that were desorbed from the kiln dust decreased as the reaction temperature was increased and the higher temperature facilitated the degradation of PCDD/Fs. However, the oxygen content, which ranged from 6–21%, had only a minor impact on the thermal reaction characteristics of PCDD/Fs. Finally, the thermal reaction characteristics of PCDD/Fs were largely affected by the origin of the kiln dust; 1.2 pg I-TEQ g^−1^ was desorbed from kiln dust originating from a cement plant that co-processed refuse-derived fuel (RDF) and 47.5 pg I-TEQ g^−1^ was desorbed from kiln dust originating from a cement plant that co-processed hazardous waste. The study also found that PCDD/F formation pathways were dependent on the origin of the kiln dust; precursor synthesis dominated PCDD/F formation on the kiln dust collected from a cement plant that co-processed RDF, while *de novo* synthesis dominated the formation of PCDD/Fs on the remaining samples of kiln dust.

## Abstract 2

Cement kiln dust is commonly recycled back into the production process.

## Graphical abstract

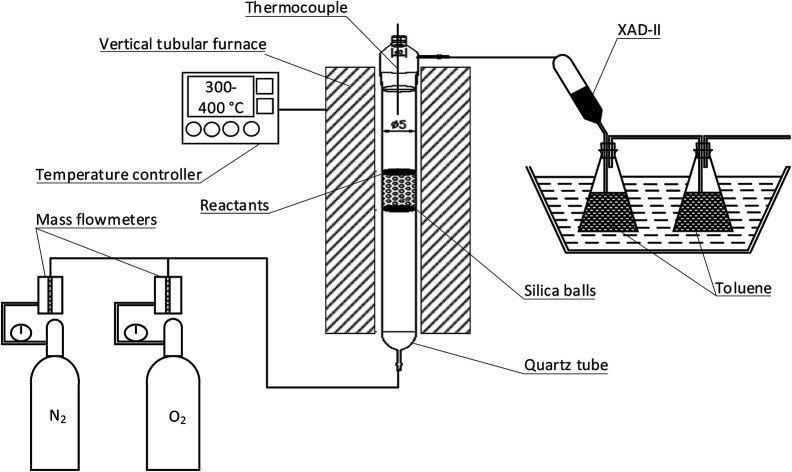

## 1. Introduction

The mass of municipal solid waste (MSW) generated in China reached 191 million tons in 2015, whereas the installed treatment capacity of the MSW incineration plants in operation throughout China was just 80 million tons.^1^ For that reason, most of the MSW generated that exceeds the installed incineration capacity is still landfilled. This has motivated researchers to focus on the development of alternative MSW disposal methods. One such method is co-processing MSW in cement kilns. The disposal method is regarded to represent a viable option for managing MSW, especially in China, where demand for cement is continually growing. Furthermore, more than 24 cement plants have acquired licenses to co-process MSW, creating a daily treatment capacity of 12 000 t of MSW.

One issue with co-processing waste in cement kilns is that polychlorinated dibenzo-*p*-dioxins and dibenzofurans (PCDD/Fs) are inevitably formed during the process of cement production.^2–4^ Moreover, airborne emissions from some cement kilns might even exceed the emission standards of 0.1 ng I-TEQ Nm^−3^ set in China.^5–7^ Therefore, significant efforts have been invested in studying the formation, destruction, and desorption characteristics of PCDD/Fs in order to reduce the PCDD/F emissions that cement plants currently generate.

The kiln dust collected from bag filters in cement plants is generally recycled into the first stage of a cyclone preheater, which results in the formation of PCDD/Fs. The recycled kiln dust acts as the basis for PCDD/Fs formation because it has relatively high contents of chlorine and carbon. Li *et al.*^8^ previously demonstrated that the first stage of a cyclone preheater is the prevailing point at which dioxins form in cement kilns; 12% of the total gaseous PCDD/Fs were generated therein. Furthermore, it is more challenging to reduce PCDD/Fs in the gas phase than it is to reduce them in the solid phase.

The expected temperature range for the *de novo* synthesis of PCDD/Fs is 250–450 °C.^11^ Meanwhile, PCDD/Fs can be degraded at temperatures of 200–600 °C.^12^ Therefore, the simultaneous formation and destruction of PCDD/Fs in the recycled kiln dust is possible during the first stage of a cyclone preheater. Furthermore, the thermal reaction characteristics of PCDD/Fs are not only influenced by their concurrent formation and degradation, but also by the initial content of PCDD/Fs in the dust. Furthermore, the temperature and oxygen content of flue gases might also influence the thermal reaction characteristics of PCDD/Fs. For instance, about 94% of the PCDD/Fs, which are contained in MSWI fly ash studied, is found in the gas phase when the reaction temperature is 350 °C.^13^ The balance between the formation and degradation effects of PCDD/Fs is largely dependent on the reaction temperature, and 450 °C is regarded as a breakthrough temperature at which the destruction of PCDD/Fs dominates their formation.^14^ Also, the physicochemical characteristics of cement kiln dust are different to those of MSWI fly ash, especially the contents of Cu and Cl.^15,16^ Furthermore, the types of waste that are co-processed in cement kilns can affect the characteristics of the kiln dust. All these factors highlight the complexity of the thermal reaction behavior of the PCDD/Fs contained in kiln dust.

Some previous studies have focused on the formation, degradation, and desorption behavior of PCDD/Fs in relation to MSWI fly ash;^9,10^ however, similar studies that focus on the kiln dust have not yet been performed. Therefore, there is a need to study the thermal reaction characteristics of PCDD/Fs in the kiln dust alongside the key factors that influence such characteristics. The present study analyzed the impact of several parameters on the thermal reaction characteristics of the PCDD/Fs present in cement kiln dust. Particularly, the reaction temperature was modified within 300–400 °C. Further, the oxygen content was changed within 6–21%. Finally, the impact of the waste co-processed in cement kilns on the thermal reaction characteristics of PCDD/Fs was studied.

To comprehensively examine the thermal reaction behavior of PCDD/Fs, both the concentrations of PCDD/Fs and the gas/particle distributions thereof were observed. The concurrent formation and degradation reactions of PCDD/Fs were analyzed by assessing the homologue and congener distributions of PCDD/Fs.

The results of the study expose the relative contribution kiln dust makes to the formation of gaseous PCDD/Fs in the flue gas in cement kilns that co-process waste. Furthermore, the results can be used to determine whether the kiln dust can be returned to other parts of cement kilns; *e.g.*, the second stage of a cyclone preheater or a precalciner, to prevent the accumulation of PCDD/Fs during the first stage of a cyclone preheater. By doing so, the emissions of PCDD/Fs could be further reduced.

## 2. Experimental

### 2.1. Laboratory set-up

**Fig. 1:**
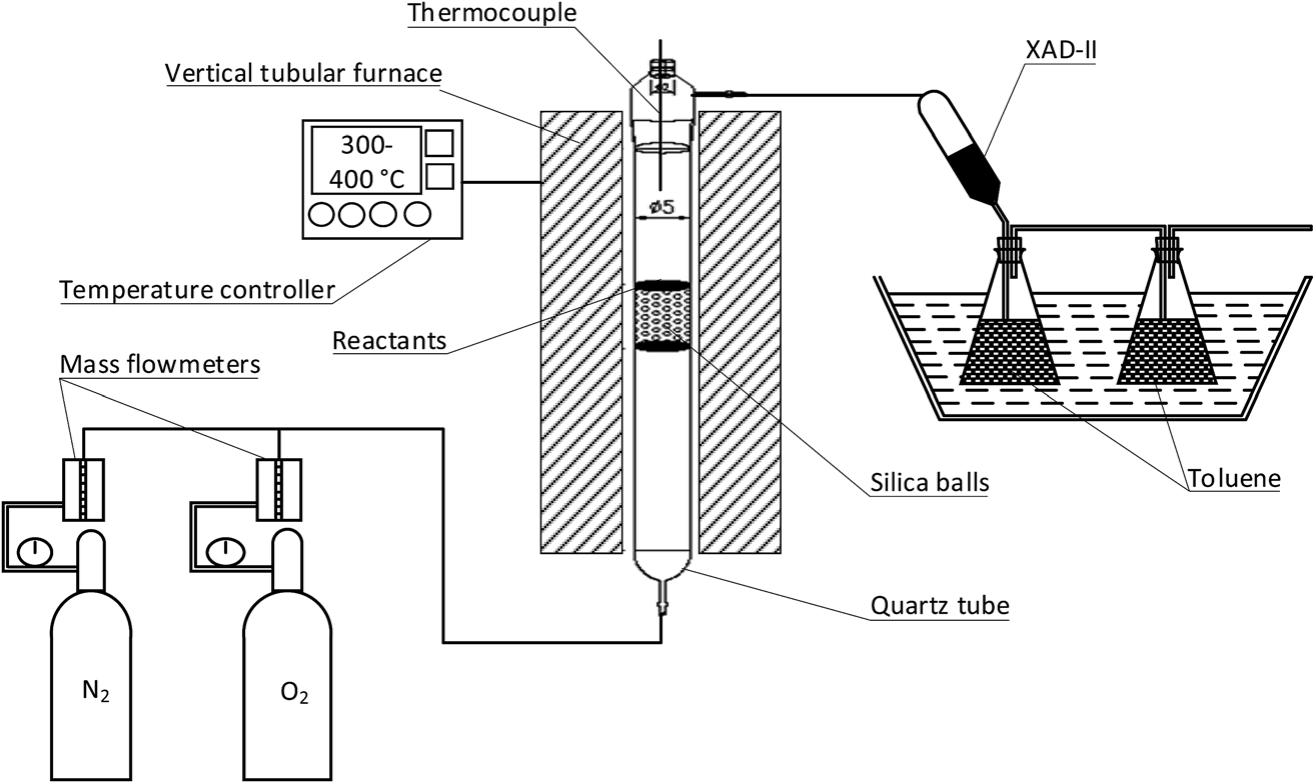
Apparatus used for the desorption experiments.

Fig. 1 illustrates the apparatus that were used to conduct the thermal reaction experiments. The apparatus comprised of a vertical tubular furnace consisting of a heated section and a temperature controller. The reaction temperature inside the vessel was simultaneously controlled with an S-type thermocouple to ensure the high accuracy of a chosen temperature regime. A quartz reactor tube (cylindrical geometry *d* = 50 mm and *l* = 530 mm) was filled with silica balls (*d* = 4 mm) up to a height of 80 mm. The silica balls were used to stabilize and homogenize the flow of a reaction gas. A quartz plate containing the reactants (kiln dust) was placed inside the quartz reactor tube right onto the silica balls at the point at which the temperature set for a specific experiment was reached and stable conditions had been achieved. The reaction gas was injected from the bottom of the reactor and was flushed through the reactants to carry gaseous PCDD/Fs to a collection zone that included XAD-II resin and toluene. The reaction gas was retained for 55 seconds.

### 2.2. Materials

One sample of kiln dust (KD1) was collected from a cement kiln that had a daily clinker capacity of 5000 t. The cement plant employs a dry production process and employs a state-of-the-art configuration with a preheater/precalciner system containing five cyclones.^8^ The kiln dust collected from a bag filter is recycled during the first stage of the cyclone preheater. The cement plant co-processes RDF, which is fed into the precalciner at a constant rate of 15 t h^−1^. Some primary characteristics of the hazardous waste co-processed in the cement kiln from which KD1 was sampled are given in Table 1.

**Table 1:** Contents of metals, water, chlorine, and heating value of RDF co-processed in the cement plant from which KD1 was sampled

	Water content, wt%	Lower heating value, MJ kg^−1^	Cu, mg kg^−1^	Cd, mg kg^−1^	Cr, mg kg^−1^	Pb, mg kg^−1^	As, mg kg^−1^	Ni, mg kg^−1^	Chlorine, wt%
RDF	37.9	5.77	149	21.4	339	387	71.7	63.8	0.136

The second sample of kiln dust (KD2) was collected from a cement kiln that had a similar configuration to that from which KD1 was collected. The second kiln had a daily clinker capacity of 2000 t. No waste was co-processed in the cement kiln.

The last sample of kiln dust (KD3) was collected from a cement kiln that had a daily capacity of 4000 t. 9 t h^−1^ hazardous waste was co-processed in this cement kiln. The waste contained pesticide waste, incineration fly ash, Cr-containing waste, and non-ferrous metal smelting waste. The characteristics of the hazardous waste that was co-processed in the cement kiln from which the KD3 sample was taken are given in Table 2.

**Table 2:** The characteristics of hazardous waste co-processed in the cement plant from which KD3 was sampled^a^

Waste	Water content, wt%	Lower heating value, MJ kg^−1^	SiO_2_, wt%	Sulfur, wt%	Fluorine, mg kg^−1^	Chlorine, mg kg^−1^	Copper, mg kg^−1^
Incineration fly ash	3.6	0.43	24.7	0.001	0.96	0.96	7.5
Cr-containing waste	13.1	0.96	1.7	0.1	0.23	4.48	1.9
Non-ferrous metal smelting waste	1.9	6.1	14.5	0.089	12.4	0.91	6.1
Ni-containing waste	33.8	0.56	11.9	0.055	0.23	0.38	3.1
Organic waste solvent	33.8	11.3	1.5	0.098	0.50	1.44	0.9
Hazardous waste	33.8	0.89	14.5	0.013	N.D.	0.95	0.4

a N.D. – not detected.

### 2.3. Design

Table 3 introduces the conditions of the experiments that were conducted in the present study. The experiments included a reference Experiment (R-0), as well as three series of experiments; namely A, B, and C, to study the impact of each chosen parameter on the desorption characteristics of PCDD/Fs. The R-0 reference experiment was conducted using KD1 at a temperature of 350 °C and oxygen content of 6%. The series A studied the influence of the reaction temperature on the desorption behavior of PCDD/Fs in the kiln dust. The reaction temperature was set to 300 °C for Experiment A-1 and 400 °C for Experiment A-2. The remainder of the parameters remained the same as those employed for the R-0 reference experiment. Series B studied the influence the oxygen content of the reaction gas had on the desorption behavior of PCDD/Fs. The oxygen content in the reaction gas was set to 10% for Experiment B-1 and 21% for Experiment B-2. Series C studied the influence the origin of the kiln dust had on the desorption behavior of PCDD/Fs; KD2 was used in Experiment C-1 and KD3 in Experiment C-2.

**Table 3:** Conditions of the conducted experiment. Values highlighted in bold represent the experiments that varied in comparison to the reference experiment

No.	Temperature	Oxygen content	Reactant
R-0	**350 °C**	**6% O**_**2**_	**KD1**
A-1	**300 °C**	6% O_2_	KD1
A-2	**400 °C**	6% O_2_	KD1
B-1	350 °C	**10% O**_**2**_	KD1
B-2	350 °C	**21% O**_**2**_	KD1
C-1	350 °C	6% O_2_	**KD2**
C-2	350 °C	6% O_2_	**KD3**

In all experiments, the flow rate of the reaction gas was set to 300 ml min^−1^, and the mass of the reactant was 8 g. Each experiment lasted 30 minutes to ensure completeness of reactions. Both the kiln dust and the gaseous compounds were collected and analyzed in parallel. Each experiment was replicated to ensure the reliability of the results.

### 2.4. PCDD/Fs analysis

The pretreatment and quantification of the collected gaseous samples was performed in accordance with the US EPA Method 1613.^17^ The pretreatment process included a Soxhlet extraction, concentration in a rotary evaporator, acid washing, cleaning on a mixed acid/basic silica gel chromatographic column, cleaning on an alumina chromatographic column, and concentration in a nitrogen flow. The identification and quantification of PCDD/Fs was accomplished by a high-resolution gas chromatography-high-resolution mass spectrometry (HRGC-HRMS) method using a 6890 Series gas chromatograph (Agilent, USA) employing a DB-5ms (60 m × 0.25 mm I.D., 0.25 μm film thickness) capillary column for separation of the PCDD/Fs congeners, and a JMS-800D mass spectrometer (JEOL, Japan). The temperature program during HRGC was optimized as follows: (a) splitless injection of 1 μl at the initial oven temperature of 150 °C, which was kept for 1 min; (b) temperature increased to 190 °C at the rate of 25 °C min^−1^; and (c) temperature increased to 280 °C at the rate of 3 °C min^−1^ with the subsequent duration of the experiment being 20 min from the point at which the temperature was reached. The mean recoveries of standards for PCDD/Fs ranged from 55–125%, which was within the acceptable range of 25–150%.

## 3. Results

### 3.1. Concentration of 2,3,7,8-PCDD/Fs

**Fig. 2:**
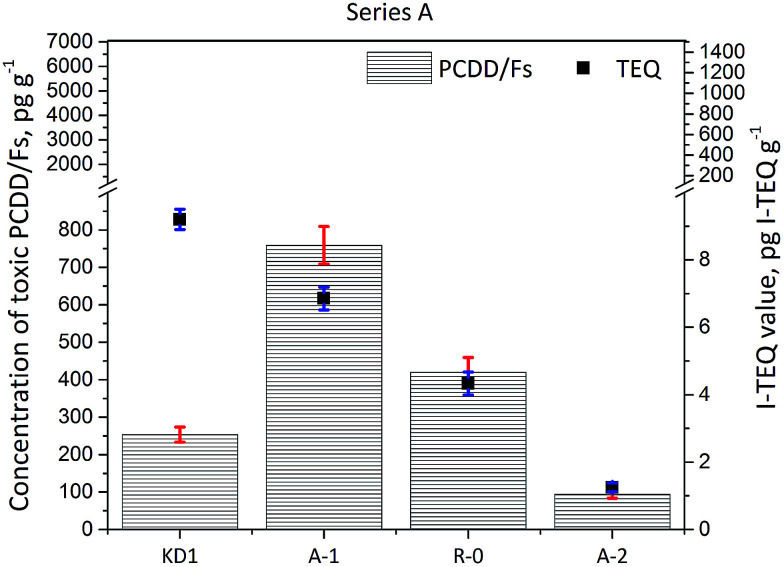
Concentrations of 17 toxic PCDD/Fs and I-TEQ values under the impact of the reaction temperature.

The concentrations of PCDD/Fs and the corresponding I-TEQ values are presented in Fig. 2. The concentration of PCDD/Fs in the KD1 and KD2 samples was 254 ± 20 pg g^−1^ (9.2 ± 0.3 pg I-TEQ g^−1^) and 1823 ± 90 pg g^−1^ (190 ± 6 pg I-TEQ g^−1^) respectively. A significantly higher concentration of PCDD/Fs of 6455 ± 500 pg g^−1^ (1399 ± 100 pg I-TEQ g^−1^) was identified in the KD3 sample. The concentration of PCDD/Fs in KD3 was at the same level as the concentration of PCDD/Fs in MSWI fly ash.^18,19^ The significantly higher mass of PCDD/Fs released from KD3 could primarily be attributed to the operation conditions of the kiln, and the configuration of the kiln where kiln dust was sampled. The characteristics of waste co-processed in cement kilns, however, have previously been found to have less influence on the formation and emissions of PCDD/Fs.^2,20^

The results of the Series A experiments are presented in Fig. 2. As the data indicate, increasing the reaction temperature reduced the concentrations of PCDD/Fs and I-TEQ values. However, higher concentrations of PCDD/Fs were observed in the R-0 and A-1 experiments than in the original kiln dust KD1. The increase in the PCDD/Fs concentration observed during Experiment R-0 and Experiment A-1 suggested the prevalence of the formation effects of PCDD/Fs over their degradation at the temperatures of 300 and 350 °C. Still, the degradation effect of PCDD/Fs was enhanced at a higher temperature of 400 °C, at which point the concentration of PCDD/Fs fell to 94 pg g^−1^, and the I-TEQ value decreased to 1.3 ± 0.1 pg I-TEQ g^−1^, resulting in a 86% reduction efficiency of the PCDD/Fs. Although the concentrations of PCDD/Fs in the R-0 and A-1 Experiment increased in comparison to KD1, the I-TEQ values consistently decreased and were 6.9 ± 0.3 pg I-TEQ g^−1^ in Experiment A-1 and 4.3 ± 0.3 pg I-TEQ g^−1^ in Experiment R-0. Such results suggest that low chlorinated PCDD/Fs are more easily degraded than highly chlorinated PCDD/Fs. This result was aligned with findings of a previous study by Yang *et al.*^21^

**Fig. 3:**
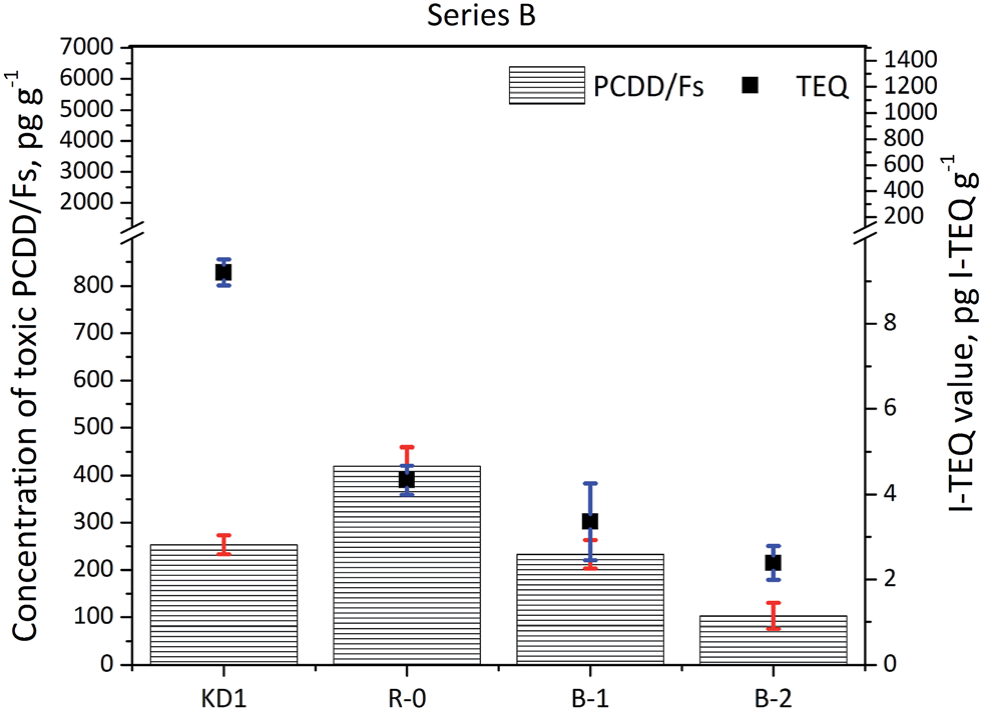
Concentrations of 17 toxic PCDD/Fs and I-TEQ values under the impact of the oxygen content.

The results of the Series B experiments are presented in Fig. 3 and show that increasing the oxygen content in the reaction gas resulted in reduced concentrations of PCDD/Fs and I-TEQ. The concentration of PCDD/Fs decreased from the reference value of 420 ± 40 pg g^−1^ (4.3 ± 0.3 pg I-TEQ g^−1^) to 233 ± 30 pg g^−1^ (3.4 ± 0.9 pg I-TEQ g^−1^) in Experiment B-1, which represented to the oxygen content increase to 10%. A further increase in the oxygen content to 21% in Experiment B-2 decreased the concentration of PCDD/Fs to 103 ± 27 pg g^−1^ (2.4 ± 0.4 pg I-TEQ g^−1^). Similarly, the study by Misaka *et al.*^22^ indicated that increasing oxygen content promotes the thermal degradation of PCDD/Fs, while Shibata *et al.*^23^ indicated that the formation of PCDD/Fs *via* the *de novo* synthesis weakens under an oxygen content higher than 10%.

**Fig. 4:**
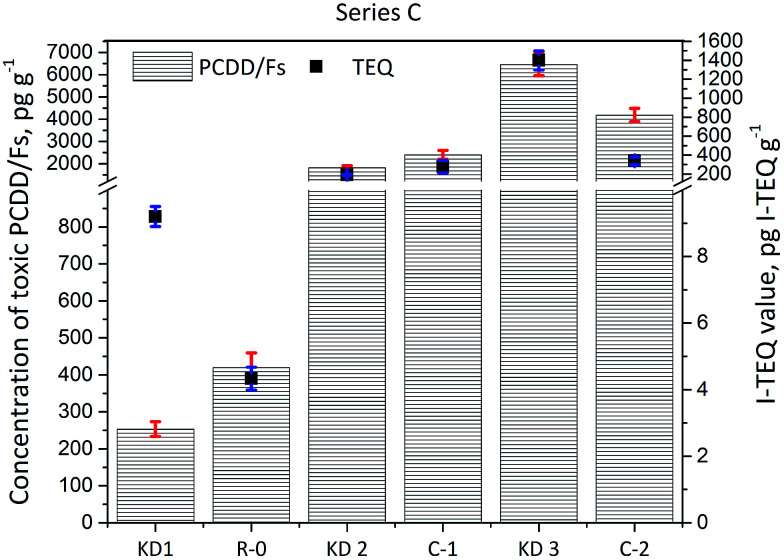
Concentrations of 17 toxic PCDD/Fs and I-TEQ values under the impact of the origin of kiln dust.

The results of the Series C experiments are presented in Fig. 4 and show that the concentration of PCDD/Fs increased from the initial 1823 ± 90 pg g^−1^ (190 ± 6 pg I-TEQ g^−1^) identified in the K2 sample of kiln dust to 2397 ± 200 pg g^−1^ (276 ± 67 pg I-TEQ g^−1^) identified in the gas phase of Experiment C-1, when KD2 was heated at 350 °C. The results indicate that PCDD/Fs are inevitably formed even without waste co-processing in cement kilns. In the case of the KD3 kiln dust, the concentration of PCDD/Fs decreased from 6455 ± 500 pg g^−1^ (1399 ± 100 pg I-TEQ g^−1^) to 4194 ± 300 pg g^−1^ (339 ± 40 pg I-TEQ g^−1^) indicating the degradation effects of PCDD/Fs were greater than the formation effects. The dominant reactions related to the PCDD/Fs in the kiln dust were determined by comparing the distribution of PCDD/Fs in the original kiln dust with the actual properties of the kiln dust.

### 3.2. Gas/particle distribution of PCDD/Fs

**Fig. 5:**
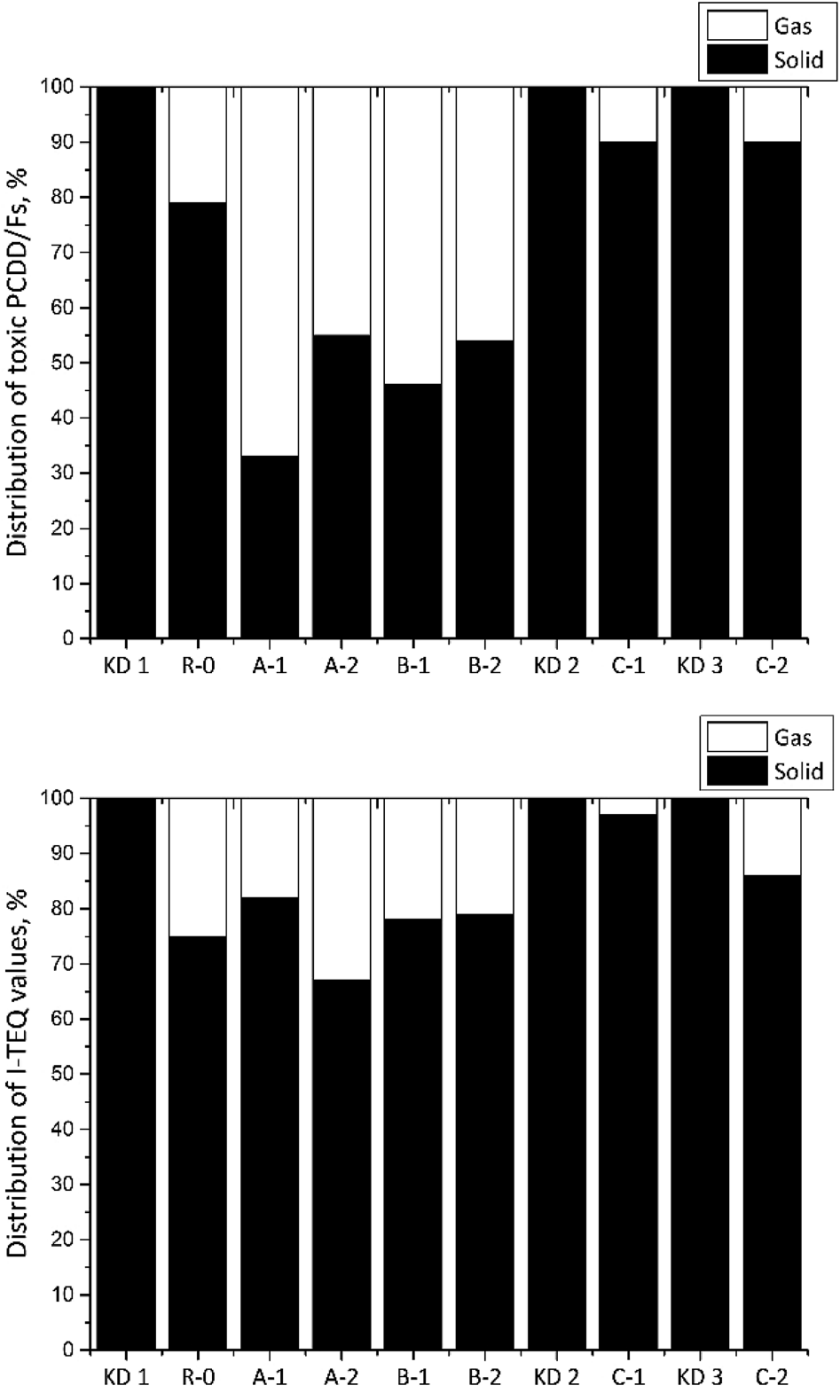
Distribution of PCDD/Fs (top) and I-TEQ values (bottom) between the gas and the solid phases.

Fig. 5 shows the distributions of 17 toxic PCDD/Fs between the gas and solid phases. Despite the fact that there was no relationship between the distribution of PCDD/Fs and the reaction temperature over the entire temperature range studied in the Series A experiments, the fraction of I-TEQ values in the gas phase increased from 18% to 33% in response to the increase in the reaction temperature from 300 °C in Experiment A-1 to 400 °C in Experiment A-2. A similar trend was found by Addink *et al.*,^24^ while the corresponding proportions of PCDD/Fs in the gas phase determined in the present study were much lower than the results achieved by Altwicker *et al.*^13^ Such differences could partly be attributed to the characteristics of the reactants and the experimental conditions. The results of the R-0 reference experiment revealed that 25% of the PCDD/Fs contained in the kiln dust was released into the flue gas. Considering the application of air pollution control devices, the impact of the PCDD/Fs originating from the kiln dust to the total emissions could be largely minimized. Furthermore, the raw meal exhibited adsorption and suppression effects on the PCDD/Fs in the flue gas.^25^

In the Series B experiments, the fractions of 17 toxic PCDD/Fs in the gas phase were 21, 54, and 47% when the oxygen contents were 6, 10, and 21% respectively. Of these, the fractions of the corresponding I-TEQ values were 25, 22 and 22%. A similar trend was observed by Addink *et al.*,^24^ indicating that the oxygen content had a minor effect on the gas/particle distribution of I-TEQ values.

In the Series C experiment, the fractions of 17 toxic PCDD/Fs in the gas phase were 10% in Experiments C-1 and C-2. However, the I-TEQ values indicated that the origin of kiln dust can affect the gas/particle distribution since only 3% of PCDD/Fs were discovered in the gas phase when kiln dust KD2 was used, which was much lower than the values for KD1 of 25% and KD3 of 13%.

### 3.3. Homologue distribution of PCDD/Fs

#### 3.3.1. Impact of reaction temperature

**Fig. 6:**
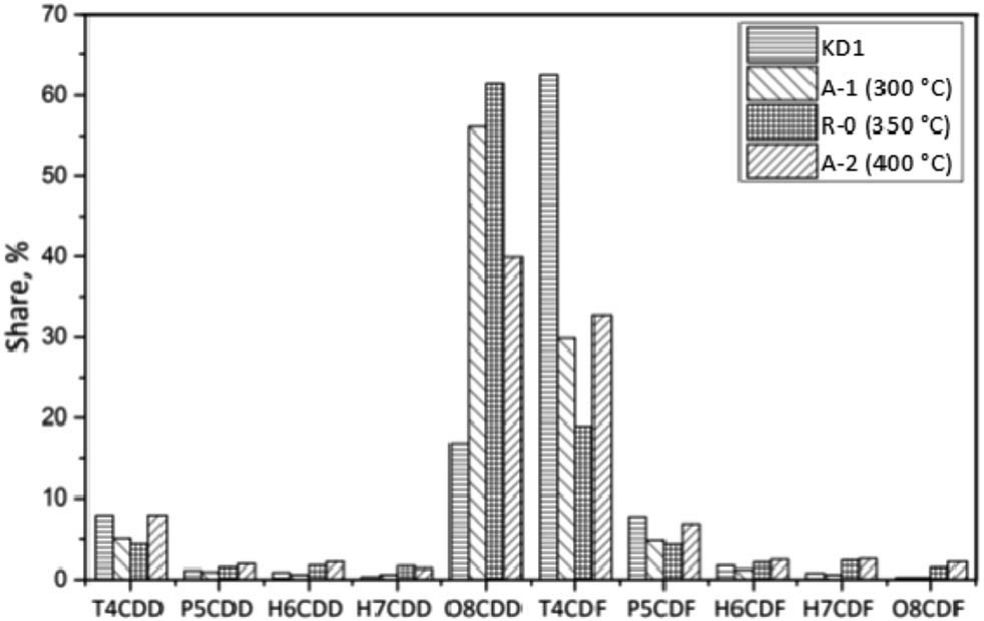
Homologue distribution of PCDD/Fs under different reaction temperatures.

Fig. 6 shows the homologue profiles of PCDD/Fs in the Series A experiments, during which the impact of the reaction temperature was studied. The fractions of polychlorinated dibenzo-*p*-dioxins (PCDDs) and polychlorinated dibenzofurans (PCDFs) in the KD1 kiln dust were 27% and 73% respectively. Tetrachlorodibenzofuran (TCDF) was the most abundant homologue, accounting for 63% of the total PCDD/Fs, which is different to that of MSWI fly ash.^26^ The total concentration of PCDD/Fs formed in the KD1 kiln dust at 300 °C (tetra- to octa-chlorinated PCDD/Fs) increased from 1210 pg g^−1^ to 1270 pg g^−1^. On the one hand, the fraction of PCDDs increased to 63% mainly due to the increase in octa-chlorodibenzo-*p*-dioxin (OCDD) from 17% to 56%. On the other hand, the fraction of TCDF decreased to 30% indicating that the chlorination and dechlorination reactions mainly occurred on the surface of the kiln dust.^27^ A higher mass of PCDDs in the gas phase in comparison to the mass of PCDFs indicated that precursor synthesis had occurred.^28^ Previous findings by Li *et al.*^29^ also demonstrated an abundance of precursors such as chlorobenzenes (CBzs) and polycyclic aromatic hydrocarbons (PAHs).

In the R-0 experiment, the concentration of PCDD/Fs decreased from 1210 pg g^−1^ to 610 pg g^−1^. The fraction of PCDDs increased to 71%. The dominant PCDD was OCDD, which accounted for 61% of the total PCDD/Fs. This indicated that OCDD were difficult to be degraded and that the precursor synthesis also contributed to the high fraction of OCDD. For Experiment A-2, the concentration of PCDD/Fs significantly decreased to 134 pg g^−1^. The fraction of PCDDs in A-2 was lower than that observed in Experiments A-1 and R-0. Moreover, the low chlorinated PCDD/Fs were relatively easily degraded due to their unstable structure. The weight average level of chlorination of PCDD/Fs increased from the original 4.86 in KD1 to 6.38 in A-1, 6.77 in R-0, and 5.98 in A-2. Such phenomenon could either be attributed to the strong combination of highly chlorinated precursors or to the degradation of low chlorinated PCDD/Fs.^30^

#### 3.3.2. Effect of oxygen content

**Fig. 7:**
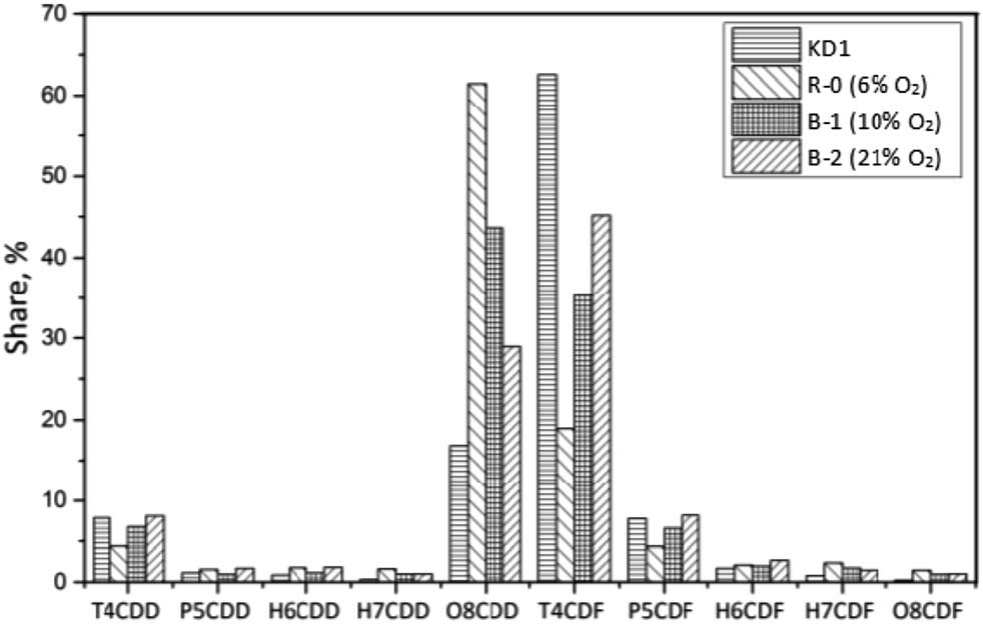
Homologue distribution of PCDD/Fs under different oxygen contents.

Fig. 7 shows the homologue profiles of PCDD/Fs in the Series B experiments, during which the impact of the oxygen content was studied. The total concentration of PCDD/Fs formed in the KD1 kiln dust decreased from 610 pg g^−1^ to 470 pg g^−1^ when the oxygen content increased from 6% to 10%. Similarly, the fraction of PCDDs decreased from 71% to 53% during the same experiments. The reason for this could be attributed to the reduction in OCDD, which was mainly formed *via* the precursor synthesis.^31^ The increase in the oxygen content decreased the weight average level of chlorination from the initial 6.38 in R-0 to 6.00 in B-1 and 5.46 in B-2, when the oxygen content increased from 6% to 10% and 21% respectively. This indicates that the dechlorinated reaction is promoted by the increase in the oxygen content.

#### 3.3.3. Different origins of KD

**Fig. 8:**
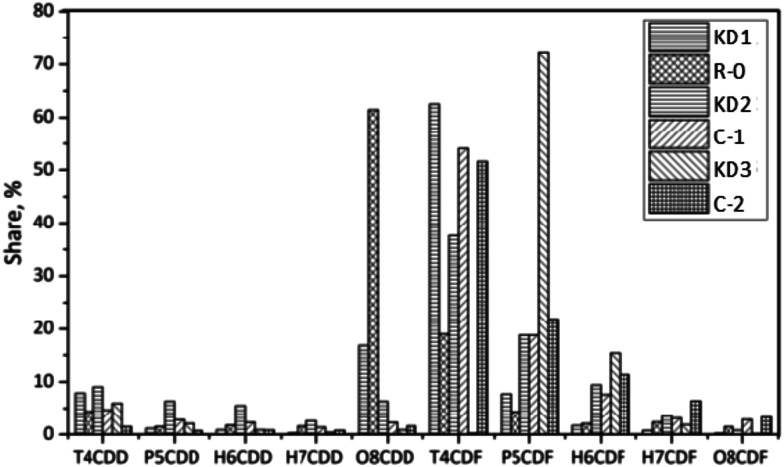
Homologue distribution of PCDD/Fs in kiln dusts of different origin.

Fig. 8 shows the homologue profiles of PCDD/Fs in the Series C experiments, in which the impact of the origin of kiln dust was studied. The total concentration of PCDD/Fs decreased from 38 900 pg g^−1^ to 23 100 pg g^−1^ for KD3. However, the total concentrations of PCDD/Fs in the original KD2 kiln dust increased from 9600 pg g^−1^ to 14 000 pg g^−1^. The differences could be attributed to the characteristics of kiln dust, including the contents of chlorine, metal catalyst, and carbon. The fractions of PCDDs in the KD1, KD2, and KD3 kiln dusts were 27%, 30%, and 10% respectively. The value increased to 71% for KD1 after the experiment, while controversial behavior was observed when KD2 and KD3 were thermally treated. This suggests that the *de novo* synthesis was the main pathway for the formation of PCDD/Fs on the KD2 and KD3 kiln dust. The dominant homologue of the KD2 was TCDF, which was the same as that of KD1. In the case of KD3, the fraction of TCDF increased from 0.3% to 52%. In the meanwhile, the fraction of pentachlorodibenzodioxin (P5CDF) decreased from 72% to 22%, indicating that the TCDF could have been formed during the dechlorination of P5CDF. The weight average level of chlorination decreased from 5.02 in KD2 to 4.76 in C-1. Similarly, the chlorination level of PCDD/Fs decreased from 5.18 in KD3 to 4.88 in C-2. The results revealed that the origin of kiln dust can significantly affect the thermal characteristics of PCDD/Fs.

### 3.4. Congener distribution of PCDD/Fs

#### 3.4.1. Effect of reaction temperature

**Fig. 9:**
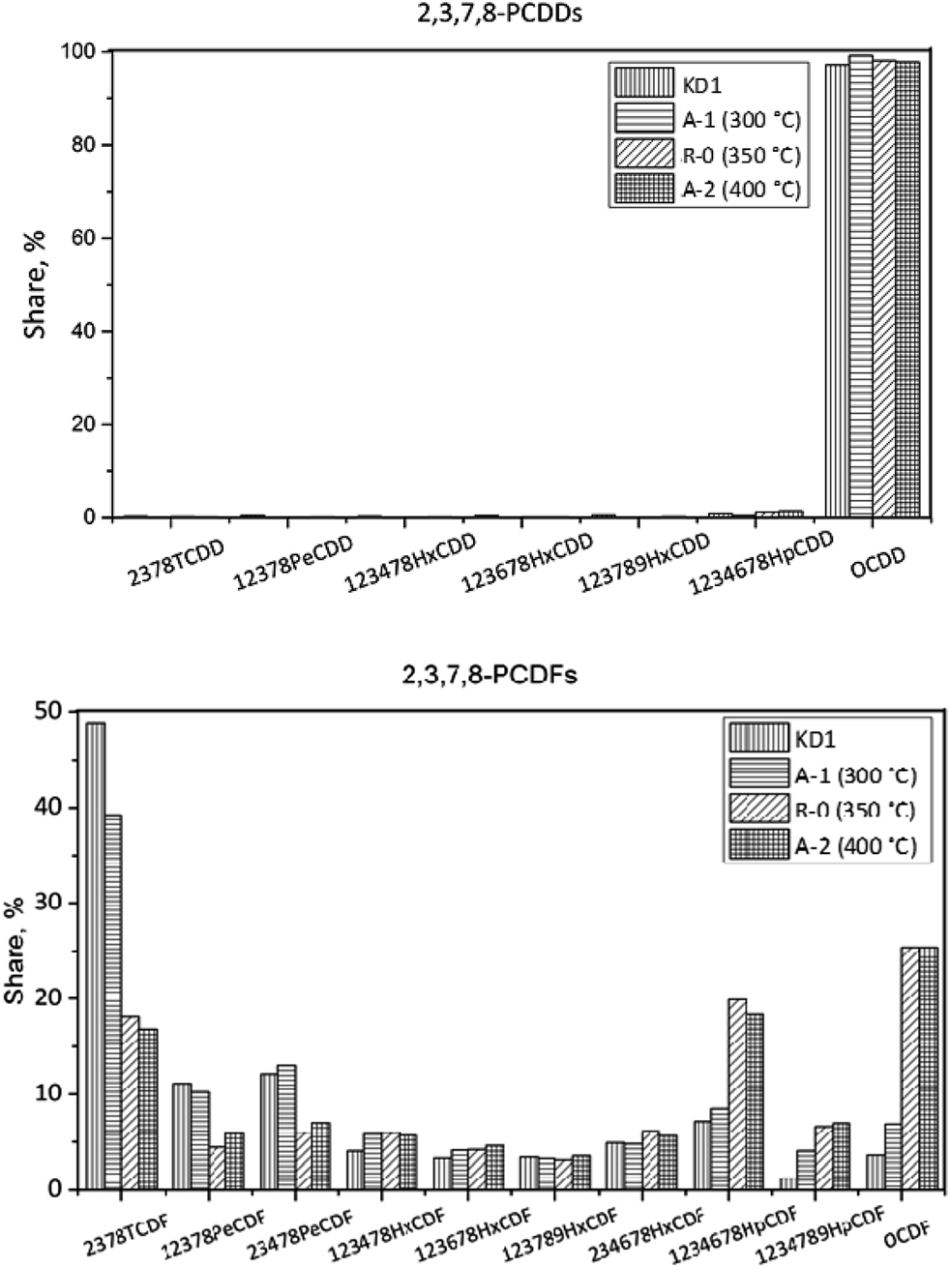
Congener distribution of PCDDs (top) and PCDFs (bottom) under different reaction temperatures.

The congener distributions of PCDDs and PCDFs under different reaction temperatures are presented in Fig. 9. The concentration of 17 toxic PCDD/Fs in the KD1 was 254 pg g^−1^, and the fraction of PCDDs was 77%. The leading PCDD and PCDF congeners were OCDD and 2,3,7,8-TCDF respectively. 2,3,4,7,8-PeCDF contributed 46% to the I-TEQ value, the most of all congeners.

In Experiment R-0, the concentration of PCDD/Fs increased to 420 pg g^−1^, while the fraction of PCDDs increased to 91%, which was dominated by OCDD. In terms of the PCDFs, the most abundant congener was also 2,3,7,8-TeCDF. In I-TEQ units, 2,3,4,7,8-PeCDF contributed 41%, the most out of all the congeners.

In Experiment A-1, the concentration of PCDD/Fs increased to 759 pg g^−1^, of which PCDDs accounted for 95%. Unlike the homologue distribution, the fraction of toxic PCDD/Fs in the gas phase was 68%. The leading PCDD and PCDF congeners in A-1 were OCDD and OCDF. Corresponding to 51%, 2,3,4,7,8-PeCDF made the most pronounced contribution to the I-TEQ value.

In Experiment A-2, the concentration of PCDD/Fs decreased to 94 pg g^−1^. The fraction of PCDDs was 88%, of which OCDD accounted for 98% and was, therefore, the most abundant congener. The most abundant PCDF congeners were 2,3,7,8-TCDF, 1,2,3,4,6,7,8-HpCDF, and OCDF which accounted for 17, 18, and 25% respectively. 2,3,4,7,8-PeCDF made the most pronounced contribution to the I-TEQ value of 46%.

#### 3.4.2. Effect of oxygen content

**Fig. 10:**
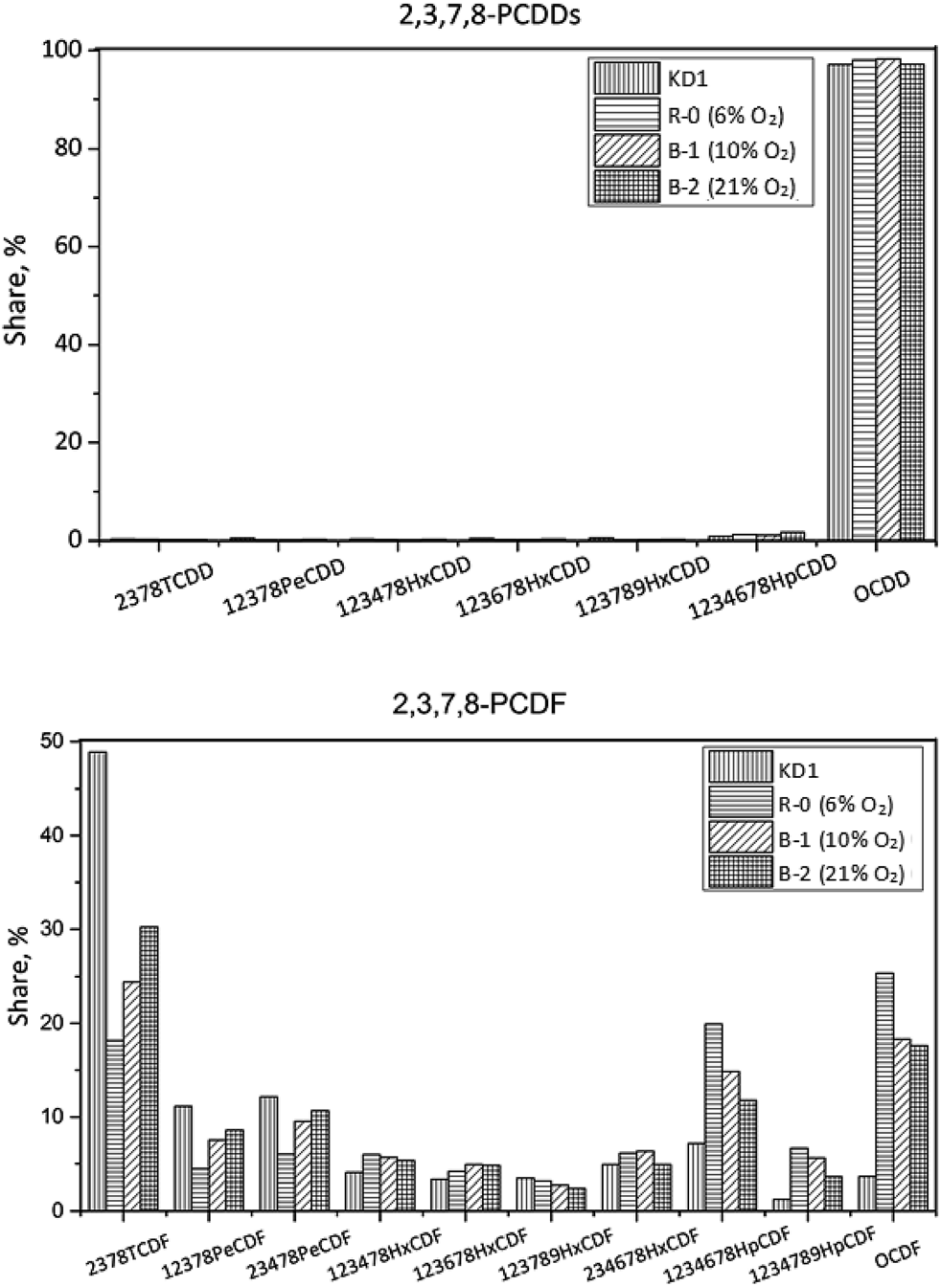
Congener distribution of PCDDs (top) and PCDFs (bottom) under different oxygen contents.

The congener profiles of PCDD/Fs under different oxygen contents are presented in Fig. 10. The concentration of 17 toxic PCDD/Fs decreased from 254 pg g^−1^ in KD1 to 233 pg g^−1^ in Experiment B-1, in which PCDDs accounted for 88% of PCDD/Fs. The leading PCDDs congener was OCDD, accounting for 98%. Similar to the results of Experiment R-0, the leading PCDF congeners in I-TEQ units were 2,3,7,8-TCDF, 1,2,3,4,6,7,8-HpCDF, and OCDF. Of these, 2,3,4,7,8-PeCDF contributed 49% to the I-TEQ value, the most out of all the congeners.

In Experiment B-2, the concentration of 17 toxic PCDD/Fs decreased to 103 pg g^−1^. The fraction of PCDDs remained the same as that of Experiment B-1, indicating that the oxygen content had a minor effect on the congener distribution of 17 toxic PCDD/Fs. OCDD was the dominant PCDD congener, accounting for 97%. As per PCDFs, 2,3,7,8-TeCDF, 1,2,3,4,6,7,8-HpCDF, and OCDF were the most abundant congeners, which reflected the outcomes of Experiments B-1 and R-0. In I-TEQ units, the contributions of each PCDD/Fs were similar to the Experiments R-0 and B-1, since their congener distributions were almost the same. The results revealed that the oxygen content had no selectivity on the desorption effect of PCDDs and PCDFs on the kiln dust.

#### 3.4.3. Different origins of KD

**Fig. 11:**
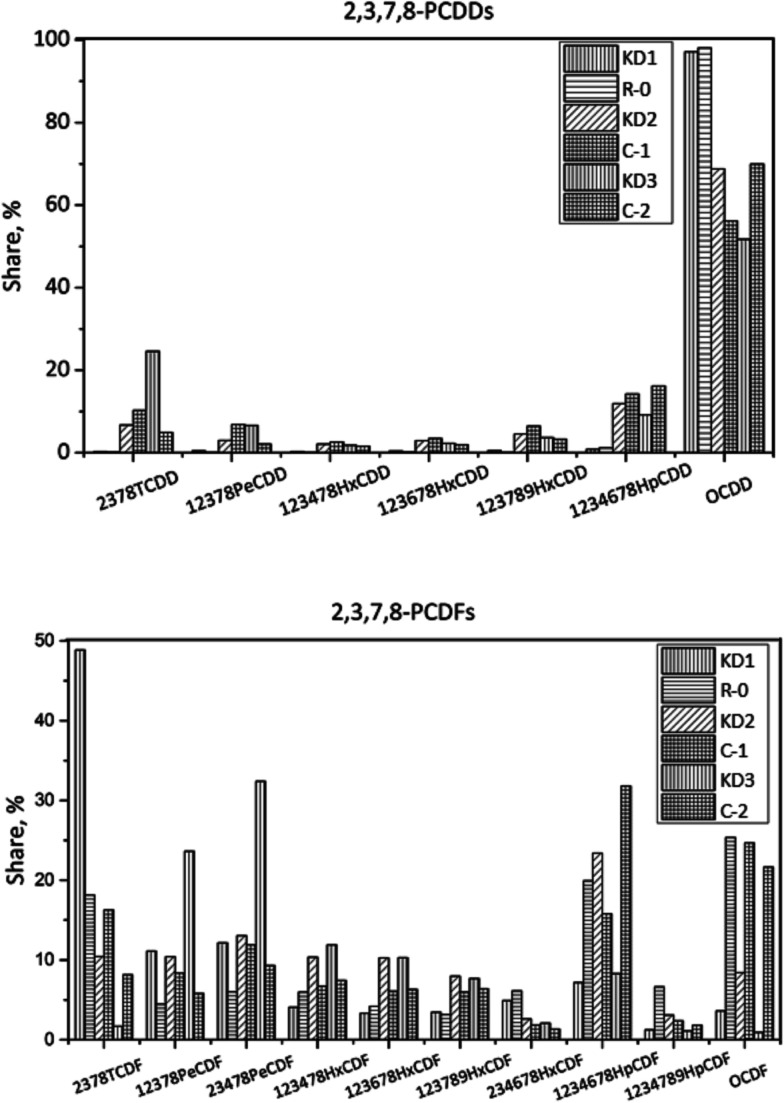
Congener distribution of PCDDs (top) and PCDFs (bottom) in kiln dusts of different origins.

The congener profiles of the 17 toxic 2,3,7,8-substituted PCDD/Fs on different origins of kiln dust are displayed in Fig. 11. The concentration of 17 toxic PCDD/Fs was 1823 pg g^−1^, of which PCDFs accounted for 51%. The leading PCDD and PCDF congeners were OCDD and 1,2,3,4,6,7,8-HpCDF respectively. In I-TEQ units, 2,3,4,7,8-PeCDF contributed 57%, the most of all congeners. In Experiment C-1, the concentration of PCDD/Fs increased to 2397 pg g^−1^, of which PCDFs accounted for 74%. The share of OCDD decreased from 69% in KD2 to 56% in C-1. In terms of PCDFs, the dominant congener was OCDF, accounting for 25%. The high accumulation of PCDD/Fs in the solid phase could be attributed to the *de novo* synthesis.^32^ Unlike other experiments, 2,3,4,7,8-PeCDF made the most significant contribution to the I-TEQ value.

The concentration of 17 toxic PCDD/Fs was 6455 pg g^−1^ in KD3, which was two times higher than that of KD2. PCDFs accounted for a substantial amount of the I-TEQ value at 90%, indicating greater *de novo* synthesis during co-processing of hazardous waste, which could supply more chlorine for the formation of PCDD/Fs. The dominant PCDD and PCDF congeners were OCDD and 2,3,4,7,8-PeCDF respectively. In terms of I-TEQ values, 2,3,4,7,8-PeCDF made the highest contribution of 78%.

In Experiment C-2, the concentration of PCDD/Fs decreased to 4194 pg g^−1^, of which PCDFs constituted 87%. OCDD was the dominant PCDD congener accounting for 70%. In terms of PCDFs, the dominant congeners were 1,2,3,4,6,7,8-HpCDF and OCDF, which accounted for 32% and 22% of PCDFs. PCDFs contributed 89% to the I-TEQ value and 2,3,4,7,8-PeCDF was the largest contributor.

## 4. Discussion

When kiln dusts KD1 and KD3 were thermally treated, the concentration of PCDD/Fs decreased from the initial values of 9.2 pg I-TEQ g^−1^ in KD1 and 1339 pg I-TEQ g^−1^ in KD3 to 4.3 pg I-TEQ g^−1^ in R-0 and 339 pg I-TEQ g^−1^ in C-2. However, an increase of PCDD/Fs concentration from the initial 190 pg I-TEQ g^−1^ in KD2 to 276 pg I-TEQ g^−1^ was observed. Such findings are in line with those of Zhan *et al.*,^28^ who studied raw meal and soxhlet fly ash and found that the concentration of PCDD/Fs increased in those cases from 3 to 55 pg I-TEQ g^−1^ for the raw meal and from 3 to 157 for the soxhlet fly at the same reaction temperature. Therefore, the origin of the kiln dust had a pronounced impact on the thermal reaction characteristics of PCDD/Fs and such an impact could be attributed to variations in the properties of the kiln dust; for example, differences in contents of chlorine or metal catalyst.

The PCDFs/PCDDs ratio of the kiln dusts KD1, KD2, and KD3 constantly exceeded 2.00. However, the same ratio decreased to 0.41 in Experiment R-0 and increased to 6.40 and 17.60 in Experiments C-1 and C-2 respectively. At the same time, a concurrent increase in the weight average level of chlorination from 4.86 to 6.77 in KD1 and concurrent decrease from 5.02 and 5.18 to 4.76 and 4.88 in KD2 and KD3 respectively, was observed. Such behavior can be explained by the higher stability of highly chlorinated PCDD/F congeners compared to the low chlorinated ones. The results indicated that the *de novo* synthesis dominated formation of PCDD/Fs in the KD2 and KD3 samples, while identifying the main formation pathway of the PCDD/Fs for KD1 kiln dust was challenging.

As Table 4 highlights, the I-TEQ concentration of PCDD/Fs in the gas phase identified during thermal treatment of kiln dust KD1 decreased from 1.2 pg I-TEQ g^−1^ in Experiment A-1 to 0.4 pg I-TEQ g^−1^ in Experiment A-2. On the other hand, the elevated temperature resulted in an increase in the share of I-TEQ identified in the gas phase from 18 to 33% for the same samples. Likewise, the increasing oxygen content resulted in a reduction in the I-TEQ concentration of PCDD/Fs in the gas phase from 1.1 pg I-TEQ g^−1^ in R-0 to 0.5 pg I-TEQ g^−1^ in B-2.

**Table 4:** Concentrations and distributions of PCDD/Fs before and after the experiments

Item	Unit	KD1	R-0	A-1	A-2	B-1	B-2	KD2	C-1	KD3	C-2
Temperature		—	350 °C	300 °C	400 °C	350 °C	350 °C	—	350 °C	—	350 °C
Oxygen content		—	6%	6%	6%	10%	21%	—	6%	—	6%
2,3,7,8-PCDDs	pg PCDDs g−1	195	382	719	82	207	85	885	619	666	536
2,3,7,8-PCDFs	pg PCDFs g−1	59	38	40	12	26	18	938	1778	5789	3658
Σ2,3,7,8-PCDD/Fs	pg PCDD/Fs g−1	254	420	759	94	233	103	1823	2397	6455	4194
Fraction of 2,3,7,8-PCDD/Fs in gas phase	%	—	21%	68%	45%	54%	47%	—	10%	—	10%
I-TEQ	pg I-TEQ g−1	9.2	4.3	6.9	1.3	3.4	2.4	190	276	1399	339
I-TEQ (gas)	pg I-TEQ g−1	—	1.1	1.2	0.4	0.7	0.5	—	8.3	—	47.5
Fraction of I-TEQ in gas phase	%	—	25%	18%	33%	22%	22%	—	3%	—	14%
PCDFs/PCDDs	—	2.70	0.41	0.58	0.88	0.87	1.41	2.35	6.40	8.77	17.6
Cl-PCDDs	Weight average level of chlorination	6.63	7.61	7.61	7.19	7.38	6.99	5.69	5.54	4.83	6.07
Cl-PCDFs		4.20	4.73	4.27	4.60	4.42	4.38	4.73	4.64	5.21	4.82
Cl-PCDD/Fs		4.86	6.77	6.38	5.98	6.00	5.46	5.02	4.76	5.18	4.88

Li *et al.*^29^ described the concentrations of gaseous PCDD/Fs in the flue gas collected at the outlets of the first and the second stages of a cyclone preheater of 101 pg I-TEQ Nm^−3^ and 22 pg I-TEQ Nm^−3^ respectively. In the present study, 1.1 pg I-TEQ of PCDD/Fs g^−1^ kiln dust KD1 was released. Assuming the rates of kiln dust and stack gas of 47 t h^−1^ and 700 000 Nm^3^ h^−1^,^8^ it was calculated that the concentration of PCDD/Fs in the flue gas at the first stage of a cyclone preheater could increase by 74 pg I-TEQ Nm^−3^ due to the desorption of PCDD/Fs from the kiln dust. Still, the actual contribution the PCDD/Fs desorbed from KD1 made to the total emissions would be lower than the calculated value of 74 pg I-TEQ Nm^−3^ due to the presence of alkaline raw materials, which can inhibit the formation of PCDD/Fs in the kiln dust.^33^ Moreover, gaseous PCDD/Fs will be abated when passing through the suspension preheater, raw mill, and bag filter as described by Li *et al.*,^29^ who reported a reduction in the PCDD/Fs concentration from 101 to 13 pg I-TEQ Nm^−3^. Therefore, as reported by Li *et al.*,^29^ PCDD/Fs originating from their desorption from kiln dust would make a minor contribution to the overall emissions of PCDD/Fs if the PCDD/Fs reduction efficiency of 87% was achieved. However, recycling kiln dust that exhibits similar properties to the KD2 and KD3 kiln dusts during the second stage of operation of a cyclone preheater, during which higher temperatures are achieved, may be recommended to effectively destroy PCDD/Fs.

## 5. Conclusions

The thermal reaction characteristics of the PCDD/Fs contained in the cement kiln dust of varying origins and under varying conditions were investigated. The results of the study suggested that:

(1) The temperature increase from 300 °C to 400 °C reduced the mass of 2,3,7,8-PCDD/Fs desorbed from the kiln dust from 1.2 to 0.4 pg I-TEQ g^−1^. Likewise, the increase in oxygen content in flue gas from 6% to 21% decreased the mass of 2,3,7,8-PCDD/Fs desorbed from the kiln dust from 1.2 to 0.5 pg I-TEQ g^−1^. This implies that treating kiln dusts at higher temperatures and in gases with higher oxygen contents enhances the PCDD/Fs degradation effect.

(2) The leading PCDD/Fs formation pathway was precursor formation on kiln dust KD1, while the *de novo* synthesis dominated the formation mechanisms of PCDD/Fs in kiln dusts KD2 and KD3.

(3) Recycling kiln dust that is similar in properties to the KD1 kiln dust during the first stage of a cyclone preheater would not significantly increase the emission of PCDD/Fs. However, recycling kiln dust that exhibits similar properties to the KD2 and KD3 kiln dusts during the second stage of the operation of a cyclone preheater, during which higher temperatures are achieved, may me recommended to effectively destroy PCDD/Fs.

## Conflicts of interest

There are no conflicts to declare.

## Supplementary Material
